# Influence of polygenic risk scores for schizophrenia and resilience on the cognition of individuals at-risk for psychosis

**DOI:** 10.1038/s41398-021-01624-z

**Published:** 2021-10-09

**Authors:** Qin He, Célia Jantac Mam-Lam-Fook, Julie Chaignaud, Charlotte Danset-Alexandre, Anton Iftimovici, Johanna Gradels Hauguel, Gabrielle Houle, Calwing Liao, ICAAR study group, Isabelle Amado, Julie Bourgin, Claire Daban-Huard, Emilie Magaud, Marion Plaze, Fabrice Rivollier, Patrick A. Dion, Guy A. Rouleau, Oussama Kebir, Marie-Odile Krebs, Boris Chaumette

**Affiliations:** 1 Université de Paris, Institute of Psychiatry and Neuroscience of Paris (IPNP), INSERM, U1266 Paris, France; 2 GHU Paris Psychiatrie et Neurosciences, Hôpital Sainte Anne, Paris, France; 3 NeuroSpin, Atomic Energy Commission, Gif-sur-Yvette, France; 4 Department of Human Genetics, McGill University, Montréal, QC Canada; 5 Department of Neurology, Montreal Neurological Institute, McGill University, Montréal, QC Canada; 6 Department of Psychiatry, McGill University, Montréal, QC Canada

**Keywords:** Schizophrenia, Comparative genomics

## Abstract

Cognitive impairment is a core feature of schizophrenia which precedes the onset of full psychotic symptoms, even in the ultra-high-risk stage (UHR). Polygenic risk scores (PRS) can be computed for many psychiatric disorders and phenotyping traits, including scores for resilience. We explored the correlations between several PRS and neurocognition in UHR individuals. We included 107 UHR individuals; 29.9% of them converted to psychosis (UHR-C) while 57.0% did not (UHR-NC) during the 1-year follow-up. Cognitive performances were assessed with the Wechsler Adult Intelligence Scale estimating the Intelligence Quotient (IQ), the Trail Making Test, the verbal fluency, the Stroop test, and the Wisconsin card sorting test. Linear regression models were used to test their association with the PRS for schizophrenia, bipolar disorder, major depression, ADHD, cross-disorders, cognitive performance, intelligence, education attainment, and resilience to schizophrenia. UHR-C had a lower IQ than UHR-NC. The PRS for schizophrenia negatively correlated with IQ, while the PRS for cognitive performance and for resilience positively correlated with IQ. PRS for schizophrenia showed a significant correlation with working memory and processing speed indices. PRS for schizophrenia showed a higher effect on IQ in UHR-NC, and UHR-NC with high PRS for schizophrenia had a similar IQ as UHR-C. Conversely, UHR-C with a high PRS for resilience performed as well as UHR-NC. Our findings suggest that cognitive deficits may predate the onset of psychosis. The genetic architecture of schizophrenia seems to impacts the cognition in UHR-NC. Cognition is also mediated by PRS for resilience.

## Introduction

Schizophrenia is a disabling psychiatric disorder emerging during adolescence and including a large range of positive symptoms, negative symptoms, disorganization of thoughts and behavior, as well as cognitive deficits. These deficits can involve several cognitive domains, including attention, working memory, verbal learning and memory, and executive functions. These deficits may pre-date the onset of schizophrenia and have been reported in early phases of the disorder, namely the ultra-high risk state (UHR) and the first episode of psychosis (FEP). The cognition seems to be impaired in UHR individuals [1–3] although only one-third of them will develop a characterized psychotic disorder such as schizophrenia after 36 months [4]. Two-third of UHR individuals will not develop psychotic disorders (namely non-converters, UHR-NC) because of resilience factors remained to be discovered. UHR-NC seem to be less impacted on cognition than those who will convert to psychosis (UHR-C) at least for verbal fluency, verbal and visual memory, and working memory [5]. Familial high risk individuals, defined as young people with familial history of schizophrenia, exhibit similar cognitive deficits as UHR, except for a higher impairment on Intelligence Quotient (IQ) [6]. While this result suggests a genetic contribution to the cognitive deficits in at-risk individuals, direct explorations of this association are scarce. In a previous candidate gene study, we reported an association between a polymorphism in *GRM7* and cognition in both FEP and UHR [7]. A genome-wide study has suggested that polygenic risk for schizophrenia is associated with cognitive deficits in a UHR cohort but the number of cognitive domains explored remained limited [8]. We hypothesize that whole-genome liability for schizophrenia may explain cognitive impairments seen in early phases of psychosis as this is the case in general population [2].

This assumption comes from early twin studies that report that variation in cognitive functioning and liability to schizophrenia share genetic factors [9]. More recently, large genome-wide association studies (GWAS) have identified polymorphisms associated with many psychiatric disorders and phenotypic traits, including schizophrenia, cognition or educational attainment [10–12]. By combining GWAS data from multiple phenotypes, polymorphisms jointly influencing schizophrenia and cognitive traits have been identified [13, 14]. The aggregation of polymorphisms identified by GWAS results in polygenic risk scores (PRS), an individual measure of genetic risk for the corresponding trait, regardless the effective status. Correlations between PRS generated from different psychiatric disorders and cognitive traits have been reported [15]. In particular, PRS has demonstrated the shared heritability of schizophrenia and cognition [16] or educational attainment [17]. PRS for cognition were shown to be significantly lower in schizophrenia cases compared to controls, whereas the PRS for schizophrenia were associated with lower general cognitive ability in the general population [16]. However, studies examining the influence of PRS for schizophrenia on cognition in patients with schizophrenia found no association with cognition [18–20]. These differential relationship between PRS for schizophrenia and cognition in general population and in schizophrenia has been confirmed by a meta-analysis [21]. Thus, PRS for schizophrenia may have different effects depending on the affected or unaffected status of the cohort.

In addition, the genetic risk for the different neuropsychiatric disorders is not associated with cognition in the same direction [15]. For instance, risk alleles for bipolar disorder, another psychiatric disorder exhibiting psychotic symptoms, have been associated with higher educational attainment [22]. A recent GWAS has explored the protective genetic factors involved in resilience to schizophrenia but PRS derived from it has never been tested with cognition [23].

We aimed at investigating the effects of different PRS on the cognition of UHR individuals. We took advantage of a longitudinal cohort followed during 1 year, with both cognitive and genotyping data. Cognition at baseline was extensively assessed, including the WAIS IQ subtests, the verbal fluency, the trail making test A and B (TMT-A and TMT-B), and the Wisconsin card sorting test (WCST). We computed the individual PRS based on the largest GWAS to date for schizophrenia, bipolar disorder, ADHD, major depressive disorder, a combination of five psychiatric disorders (cross-disorder) and resilience for schizophrenia. We tested the difference between UHR-C and UHR-NC on the cognitive deficits as well as with the association with PRS.

## Methods

### Population

We consecutively enrolled 134 UHR individuals in the prospective multicenter cohort ICAAR (“Influence du Cannabis sur l'émergence de symptômes psychopathologiques des Adolescents et jeunes Adultes présentant un état mental à Risque”, 2009–2014). An additional recruitment of 23 UHR with the same clinical and biological assessment was provided through the ongoing PsyDev cohort (“Etude familiale et génétique des aspects développementaux des maladies psychiatriques”). The study was approved by the institutional ethics committees (Comité de protection des personnes, Ile-de-France III, Paris, France for ICAAR and Comité de protection des personnes, Ile-de-France IV, Paris, France for PsyDev) and was carried out according to the Declaration of Helsinki. Written informed consent was obtained from all participants or their legal representatives.

Inclusion criteria were an age <30 years old, alterations in global functioning (Social and Occupational Functioning Assessment Scale score <70) during the past year, related to psychiatric symptoms and/or subjective cognitive complaints. Exclusion criteria included manifest symptoms of psychosis (fulfilling DSM-IV criteria), or other established psychiatric diagnoses (pervasive developmental disorder, bipolar disorder, obsessive compulsive disorder), serious or non-stabilized somatic and neurological disorders, head injury and IQ below 70. Non-French-native speaking individuals were excluded from the cognitive assessment. Non-European individuals were excluded from the genetic assessment.

All subjects were examined with the Comprehensive Assessment for at-risk mental state (CAARMS) [24], in its translated French version [25] by specifically trained psychiatrists followed by a consensus meeting for best-estimate diagnoses. Individuals fulfilling the criteria for at-risk mental state were characterized as UHR and followed for 1 year. Conversion to psychosis was characterized using the CAARMS-defined psychosis onset threshold. Among the 107 UHR individuals with both genetic and cognitive data, 32 of them subsequently developed a characterized psychotic disorder (converters, UHR-C) and 61 did not (non-converters, UHR-NC); the final status was unknown for 14 individuals.

### Cognitive assessment

**Fig. 1:**
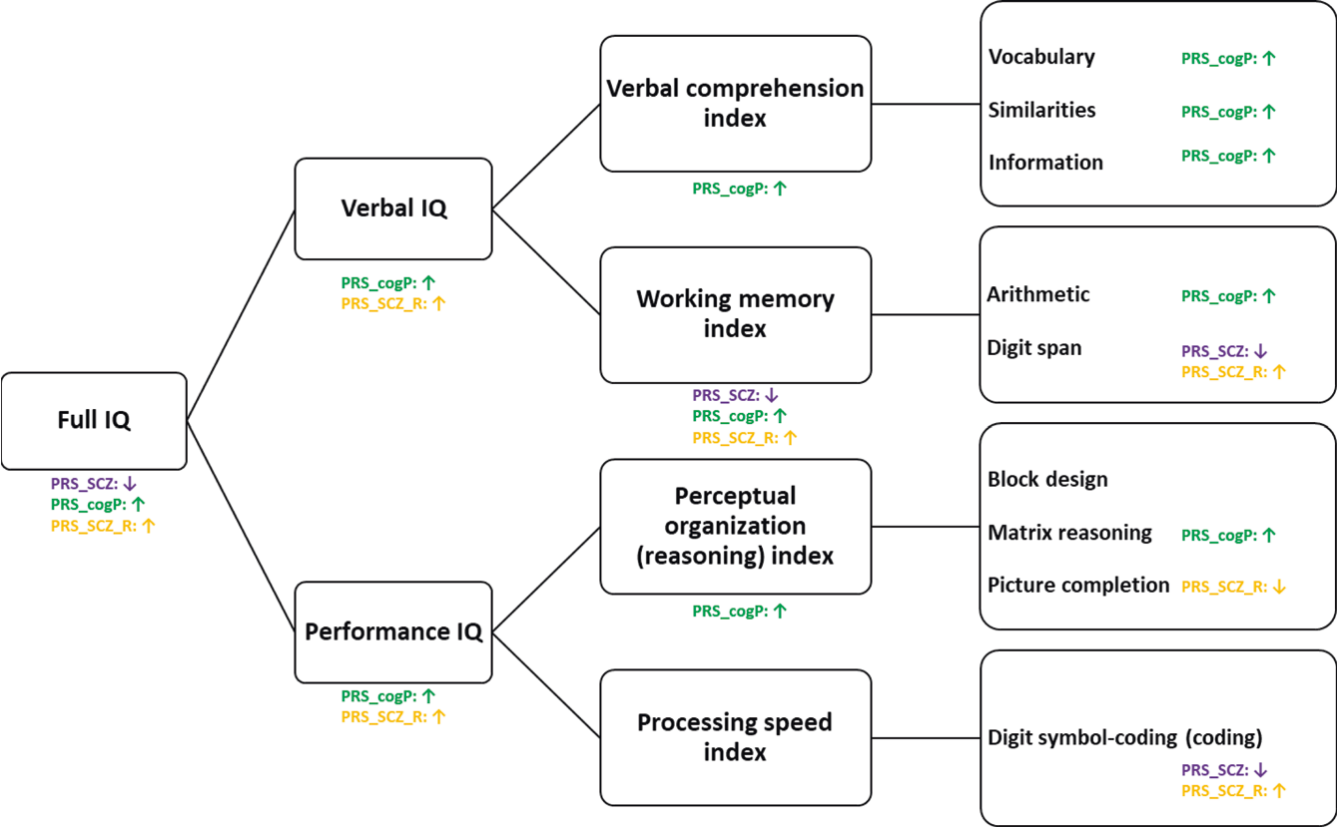
Effect of the three PRS on the WAIS subtests. Note: The significant association with PRS of schizophrenia, cognitive performance and resilience are shown in color codes (purple, green and yellow respectively). The arrow direction ↓ implicates a negative correlation, while ↑ implicates a positive correlation.

All cognitive assessments were conducted by licensed and trained neuropsychologists during face-to-face interviews as previously reported [26]. The IQ was measured at baseline by the Wechsler Adult Intelligence Scale (WAIS) for each individual. The third and the fourth versions of the WAIS were used for the ICAAR and PsyDev individuals respectively. The subtests overlapping between the WAIS III and the WAIS IV were retained for further exploration, including Vocabulary, Similarities, Information, Arithmetic, Digit Span, Block Design, Matrix Reasoning and Digit Symbol-Coding. From WAIS III to IV, the Picture Completion subtest as part of perceptual organization was deprecated. The verbal IQ, the performance IQ (also called non-verbal IQ) and four indices (verbal comprehension index, working memory index, perceptual organization index, processing speed index) were derived as shown in Fig. 1. Individuals underwent other cognitive tests: the verbal fluency [27], the D2 test of attention [28], the Stroop test [29], TMT-A and TMT-B [30], and WCST [31]. These tests were normalized by age and sex as recommended in each manual.

### Whole-genome genotyping data

Genotyping data was generated for all individuals using the genome-wide genotyping array Infinium PsychArray (Illumina, San Diego, California, United States). We performed quality control with PLINK (v1.9, www.cog-genomics.org/plink/1.9/) [32] on the raw genotyping data to exclude single nucleotide polymorphisms (SNPs) with a minor allele frequency <2%, genotyping missing rate >5% and Hardy–Weinberg Equilibrium *p* value < 10^−6^. Only autosomal SNPs were kept. The software Peddy was used to compute and check sex, relatedness and ancestry [33]. After filtering, 107 UHR individuals were confirmed with European ancestry and all passed the quality filtering. To maximize the overlap between our data and the SNPs reference panel, we did imputation using the Sanger Imputation Service (https://imputation.sanger.ac.uk/) with a pre-phase by EAGLE2 and an imputation based on a combined reference panel of UK10K + 1000 Genomes Phase 3. Imputed SNPs with an information score > 0.8 were kept. The PRS for each individual was calculated using PRSice (v2.2.8) [34] with the summary statistics of latest GWAS related to schizophrenia [10], bipolar disorder [35], major depressive disorder [36], attention deficit/hyperactivity disorder [37], and the cross-disorder GWAS of eight major psychiatric disorders [38], freely available online on the website from the Psychiatric Genomics Consortium (PGC, https://www.med.unc.edu/pgc/results-and-downloads). We also included the results from the latest GWAS on intelligence (estimated from the latent G-factor) [11], education attainment [12] and a subset GWAS of cognitive performance from the education attainment GWAS. Finally, we used the summary statistics from the schizophrenia resilience GWAS [23]. PRS was calculated using 14 *P* value thresholds (1, 0.5, 0.4, 0.3, 0.2, 0.1, 0.05, 0.01, 0.001, 1e−4, 1e−5, 1e−6, 1e−6, 1e−7, 1e−8) and we chose the best-fit threshold in linear regression for each GWAS. Clumping was performed to remove the SNPs in LD by including the 503 European samples from 1000 genome projects as reference [39]. Linear regression models were used to determine the relationships between the full IQ (FIQ) and each PRS, adjusted on age, sex and ten top principal components that reflects population structure identified as covariates by PLINK. The relationships between the subtests and the other cognitive measures were tested only for PRS significantly correlated with FIQ. PRS and cognitive measures were normalized using R to have a mean of 0 and a standard deviation of 1, in order to standardize the effect-size estimates. Thus, these estimates correspond to the standard deviation change in cognitive measures for 1 standard deviation change in PRS. Multiple-testing was corrected using the false discovery rate method (FDR, Benjamini–Hochberg method). For the association between PRS and FIQ, we corrected for the 9 PRS tested. For the association between significant PRS and the subtests and the other cognitive measures we corrected for 21 measures for each PRS. For the comparison of the slope with the null value, we performed regression tests separately in each group (UHR-NC and UHR-C). The statistical analyses were performed using R 3.6.0, and the plots were generated using “Seaborn” module in Python 3.

## Results

The future converters (UHR-C) showed a significant lower IQ at baseline than the future non-converters (UHR-NC) (Mean difference = −8 points of IQ; Student *T* test; *P* = 0.01, Table 1), although the selected GWAS were not able to predict the conversion to psychosis in UHR individuals (Supplementary Table 1, uncorrected *P* = 0.086 for PRS for schizophrenia) [40]. Among the individuals with an IQ below 90, UHR-C were more represented than UHR-NC (25% versus 14%). UHR-C and UHR-NC exhibited differences in the verbal IQ (*P* = 0.04), in the verbal communication index (*P* = 0.04), in the working memory index (*P* = 0.01) and in the TMT B minus A (*P* = 0.01).

**Table 1:** Summary statistics of the cognitive tests in the UHR samples by conversion status.

	# samples	UHR-C	UHR-NC	Two-sample comparison^a^	
	(*N* = 93)	(*N* = 32)	(*N* = 61)		
	with values	Mean ± SD	Mean ± SD	*t*	*P*
*Demographics*					
Male	56	25	31	5.44	**0.02**
Female	37	7	30		
Age	93	19.7 ± 2.6	21.9 ± 3.6	−3.01	**0.003**
*Baseline symptoms*					
BPRS	93	52.5 ± 11.5	54.1 ± 12.8	−0.60	0.55
MADRS	93	21.8 ± 8.9	21.8 ± 10.4	0.03	0.98
PANSS	93	70.4 ± 14.8	68.7 ± 17.8	0.47	0.64
SOFAS	93	46 ± 9.6	46.6 ± 9	−0.30	0.76
*WAIS scales*					
FIQ	92	98.3 ± 12.8	106.3 ± 13.9	−2.70	**0.01**
PIQ	81	95.6 ± 14.1	100.9 ± 13.2	−1.70	0.09
VIQ	80	100.2 ± 11.7	106.5 ± 13.2	−2.11	**0.04**
*Indices scores*					
Verbal communication index	90	105.9 ± 13.8	112.6 ± 14.5	−2.08	**0.04**
Working memory index	47	93.5 ± 14.1	106 ± 16.6	−2.73	**0.01**
Perceptual organization index	90	97.8 ± 12.4	103.1 ± 13.5	−1.80	0.07
*Subtests scores*					
Arithmetic	90	9.5 ± 2.2	10 ± 3.3	−0.83	0.41
Coding	90	8.9 ± 3.1	9.5 ± 3.3	−0.85	0.40
Picture completion	78	10 ± 2.8	10.8 ± 2.3	−1.46	0.15
Block design	90	9.6 ± 3	10.4 ± 3.5	−1.00	0.32
Information	90	10.4 ± 2.8	11.6 ± 3.2	−1.76	0.08
Matrix reasoning	90	9.6 ± 2.9	10.5 ± 2.9	−1.44	0.15
Digit span	90	8.9 ± 3.3	9.9 ± 3.4	−1.25	0.22
Similarities	90	11.2 ± 3.1	12.3 ± 2.7	−1.68	0.10
Vocabulary	90	11.7 ± 2.5	12.8 ± 3.1	−1.71	0.09
*Other cognitive tests*					
D2 attention	48	99.3 ± 8.5	101.5 ± 9.3	−0.84	0.40
Verbal fluency semantic animals	89	−0.4 ± 0.9	−0.2 ± 0.6	−1.37	0.17
Verbal fluency phonologic letter p	89	0.4 ± 1.1	0.6 ± 0.9	−0.85	0.40
TMT B minus TMT A	90	1.6 ± 1.6	0.8 ± 1.3	2.63	**0.01**
Stroop Temps Inter Deno	47	0.4 ± 1.3	0.1 ± 1.3	0.75	0.46
WCST percentage perseverative errors	92	51.7 ± 8.5	53.2 ± 10.3	−0.72	0.48

N: samples with non-missing values UHR-C: ultra-high risk individuals who will convert to psychosis UHR-NC: ultra-high risk individuals who will not convert to psychosis.

Symptoms at baseline including: *BPRS* brief psychiatric rating scale use to measure psychiatric symptoms such as depression, anxiety, hallucinations and unusual behavior, *MADRS* Montgomery Asberg depression rating scale self-assessment, *PANSS* Positive and Negative Syndrome Scale, and *SOFAS* social and occupational functioning assessment *scale*.

*SD* standard deviation.

^a^The comparison are based on *T*-test, except for sex comparison which used chi-square test. P values < 0.05 for these test are highlighted in bold.

**Fig. 2:**
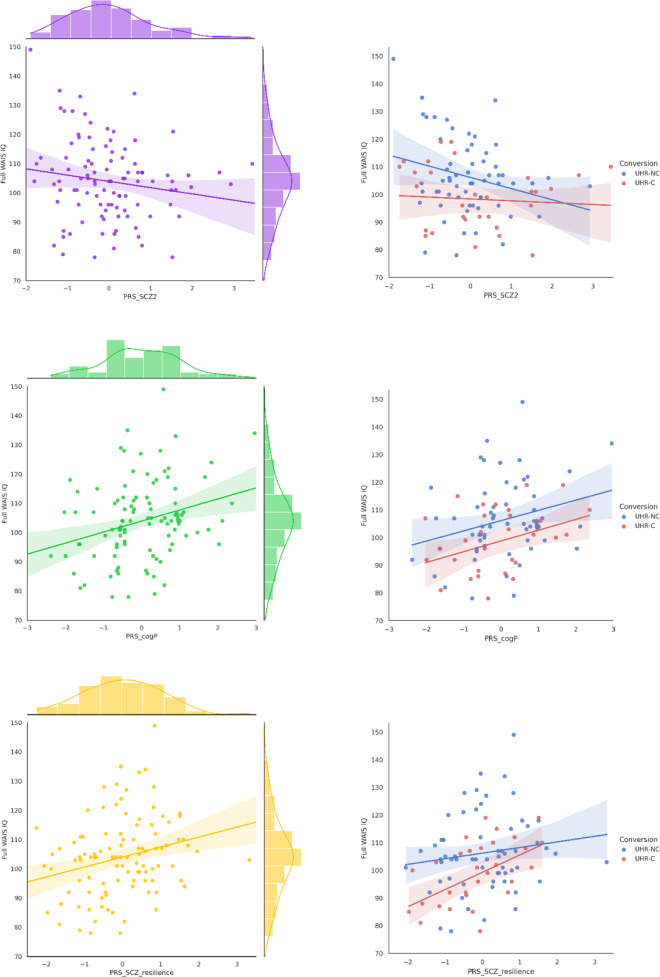
Linear regression between full WAIS IQ and PRS of schizophrenia, cognitive performance and resilience to schizophrenia. PRS_SCZ2: PRS derived from schizophrenia GWAS; PRS_cogP: PRS derived from GWAS of cognitive performance, PRS_SCZ_resilience: PRS derived from GWAS of resilience to schizophrenia.

In the whole population of UHR, we observed a significant negative relationship between the FIQ and the PRS for schizophrenia (*P* = 0.048, effect size (ES) = −0.296) (Table 2; Fig. 2). On the contrary, the FIQ related significantly and positively with the PRS for cognitive performance (*P* = 0.009, ES = 0.349) and with the PRS for resilience to schizophrenia (*P* = 0.014, ES = 0.350). The PRS derived from other major psychiatric disorders, intelligence in the general population and educational attainment were not significantly associated with FIQ. The results across the tested GWAS *P* value thresholds for all selected GWAS were shown in *Supplementary Fig.* 1. Since the FIQ follows a normal distribution with a mean of 100 and a standard deviation of 15 in the general population, we performed a sensitivity analysis by excluding 1 outlier with IQ > 145 (+3 SD); this sample is a non-converter. The effect of PRS for resilience and cognitive performance remained significant and there was a trend to significance for the PRS for schizophrenia (Supplementary Table 2). In addition, we also used a new method by computing the first PC over the set of PRS under our 14 selected thresholds [41], and we still detected a significant effect of PRS for cognitive performance with IQ (*p* = 0.001).

**Table 2:** Linear regression of full WAIS IQ on PRS adjusted on age, sex and population structure.

**FIQ ~ PRS GWAS**	**GWAS SS**	**P-val-T**	ES*	SE*	***R***^2^	**R**^**2**^a*	***P***	**FDR P**
Schizophrenia	150 064	0.1	−0.296	0.120	0.169	0.049	**0.016**	**0.048**
Schizophrenia resilience	66 617	0.01	0.350	0.114	0.197	0.081	**0.003**	**0.014**
ADHD	55 374	1.0E−07	0.136	0.104	0.129	0.003	0.197	0.222
Bipolar disorder	51 710	0.1	−0.250	0.190	0.130	0.004	0.191	0.222
Major depression	480 359	1.0E−05	−0.215	0.113	0.147	0.024	0.061	0.110
Cross-disorder 2	727 126	1.0E−03	0.073	0.108	0.117	-0.010	0.500	0.500
Intelligence	269 867	1.0E−04	0.198	0.104	0.147	0.024	0.060	0.110
Educational attainment	766 345	1.0E−06	0.178	0.101	0.142	0.018	0.081	0.122
Cognitive performance	257 828	0.3	0.349	0.102	0.216	0.102	**0.001**	**0.009**

GWAS SS: discovery sample size of the GWAS.

P-val-T: best fit *p* value threshold.

ES: effect size (coefficient in the linear regression model after stardardizing the variables).

R^2^a: adjusted R square, the variance explained by PRS. P values and corrected P values (FDR P) are highlighted in bold if they are < 0.05.

*ADHD* attention deficit hyperactivity disorder, *SE* standard error.

**Fig. 3:**
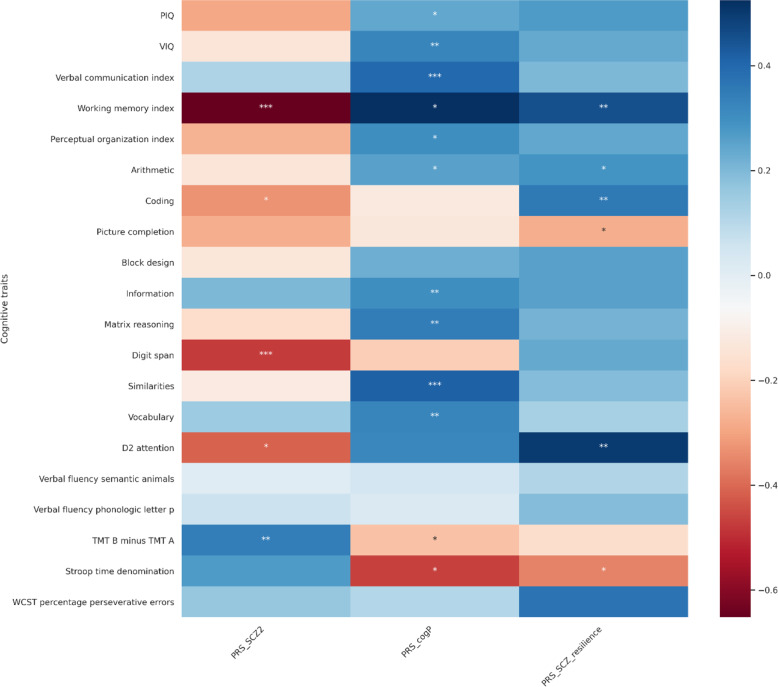
Heatmap of standardized effect size of three PRS on cognitive traits. *p < 0.05; **p < 0.01; ***p < 0.001 PRS_SCZ2: PRS derived from schizophrenia GWAS; PRS_cogP: PRS derived from GWAS of cognitive performance, PRS_SCZ_resilience: PRS derived from GWAS of resilience to schizophrenia; PIQ: performance IQ; VIQ: verbal IQ The color code refers to the coefficient of correlation (r).

We explored the relationship between the three PRS significantly associated with FIQ (PRS for schizophrenia, cognitive performance, resilience) and the scores at the index and subtests from the WAIS as well as other cognitive tests (Fig. 1; Fig. 3**;** Supplementary Table 3). PRS for cognitive performance was positively correlated with most of the cognitive measures, accordingly as a positive control. The direction of three tests are opposite compared to others (TMT B minus TMT A, Stroop time denomination and WCST percentage perseverative errors) in which the larger the value is, the lower the cognitive function (*Supplementary Fig.* 2). The working memory index was explained by the three PRS. The cognitive flexibility measured by the TMT was impaired by PRS for schizophrenia and improved by PRS for cognitive performance. In addition, PRS for schizophrenia and PRS for resilience to schizophrenia had opposite effect on attentional test (D2 test), and processing speed (Coding subtest). The strongest negative effect of PRS for schizophrenia related to digit span, an item from the working memory index.

We present the correlation between each PRS and the FIQ for the whole cohort and for UHR-C and UHR-NC respectively (Fig. 2). A low PRS for schizophrenia is associated with a significantly higher FIQ in UHR-NC than UHR-C (*P* = 0.02 for intercept comparison). The higher the PRS for schizophrenia is, the lower is the FIQ in UHR-NC (*P* = 0.049 for comparison of slope with the null value); for extreme value of PRS, the FIQ of UHR-NC is similar to UHR-C. This explains why some UHR-NC may exhibit low functioning and cognitive impairment, similar to UHR-C, even they did not develop a psychotic disorder. On the contrary, UHR-C exhibit lower IQ than UHR-NC but the PRS for schizophrenia did not seem to influence their IQ (*P* = 0.07 for comparison of slope with the null value). By comparison, the PRS for cognitive performance influence the FIQ in UHR-C and UHR-NC in a similar way (*P* = 0.642 for comparison of the slope between the two groups). Finally, PRS for resilience to schizophrenia have a positive effect on FIQ for UHR-C with those harboring a high PRS being in a similar IQ range as UHR-NC. This could explain why cognitive functioning could be preserved in a fraction of patients developing schizophrenia.

## Discussion

In this study, we explored the relationship between numerous cognitive measures and several PRS in a cohort of UHR individuals. Several cognitive deficits predate the onset of psychosis. Prior to the FEP, the UHR-C showed a lower FIQ, a poorer verbal reasoning, a lower working memory and more impairment in cognitive flexibility than UHR-NC. This replicates previous findings as it has been demonstrated that UHR-C had a worse cognition than UHR-NC [5] and that a lower IQ increases the risk for schizophrenia [2].

The FIQ is significantly explained by PRS for schizophrenia, PRS for cognitive performance, and PRS for resilience to schizophrenia with a non-negligible portion of variance explained (~5%, 8 and 10% respectively). Poorer results in working memory and cognitive flexibility are related to the PRS for schizophrenia, suggesting a direct association between the risk for the disease and the cognitive functioning in these domains. The PRS for schizophrenia is also associated with processing speed and attention. Thus, in our sample, PRS for schizophrenia is related with cognitive domains frequently reported as impaired in schizophrenia and UHR. In line with our results, the NAPLS cohort reported that the PRS for schizophrenia was modestly correlated with verbal memory (*R*^2^ = −0.14, *p* = 0.04, Hopkins Verbal Learning Test–Revised, sum of trials 1–3), and information processing speed (*R*^2^ = −0.13, *p* = 0.04, Brief Assessment of Cognition in Schizophrenia, symbol coding test) in UHR [8]. On the contrary, it has been reported that PRS for schizophrenia did not affect cognition in patients suffering from schizophrenia but in the general population. This apparent discrepancy might be explained by phase-specific effect of PRS on cognition. However, PRS for schizophrenia has not been associated with lower scores on the Wide-Range Achievement Test deemed to measure premorbid intelligence [19]. In our present study, we reported a differential effect of PRS for schizophrenia in UHR-C and UHR-NC. As UHR-NC are individuals who will not develop psychosis, they are closer to the general population and their cognition may be influenced by PRS for schizophrenia. At the same time, the genetic background of UHR-C is similar to patients with schizophrenia and consequently may not be under the influence of the PRS for schizophrenia. This could be one of the reasons why UHR exhibit a heterogeneous cognitive profile depending on their clinical outcome and the potential effect of their PRS. The reasons why the PRS for schizophrenia impacts the cognition in unaffected individuals but not in patient with schizophrenia is not fully understood. In their large cohort of patients with schizophrenia, Richards et al. found that the genetic variation in cognitive performance in schizophrenia is essentially driven by factors that influence cognition in the general population, but no evidence of association with liability to MDD or BPD or schizophrenia [18]. They concluded that mechanisms of cognitive variation within schizophrenia are at least partly independent from those that predispose to schizophrenia diagnosis itself. We might hypothesize that PRS for schizophrenia contributes to the risk for developing the disease, and that the disease onset will decrease the cognitive abilities by itself. This would explain why UHR-C have higher cognitive deficits than UHR-NC. With this floor effect, PRS for schizophrenia may not be able to influence the cognition in UHR-C and in patients with schizophrenia. However, PRS for cognition and PRS for resilience to schizophrenia may play a protective role in the cognition of UHR-C.

The genetic of resilience to schizophrenia is a new concept following the release of a GWAS that explored heritable variation promoting resistance to disease by reducing the penetrance of risk loci. It has been constructed by conducting a GWAS on unaffected individuals carrying a high PRS for the disease. With this approach, resilience alleles are not simply the inverse of the risk-associated alleles. In our cohort, PRS for resilience to schizophrenia mitigate the effect of PRS for schizophrenia on working memory, processing speed, and attention. However, we could not detect any effect on cognitive flexibility.

These results highlight the interest of working on early phases of psychosis. UHR are less exposed to confounders than schizophrenia: they are less medicated and the shorter symptomatic duration decreases the potential toxicity of the illness. Moreover, the results challenge the categorical diagnosis. Some UHR-NC are as impaired as UHR-C on their cognition without developing the disease. Rather than focusing on conversion to psychosis, the identification of factors influencing the functional outcome and dimensional phenotypes would be of great interest.

Our study has several limits. First, our cohort is small and we may have a lack of power, especially to determine the association between FIQ and PRS for intelligence and educational attainment. Even we did not report a significant association, the coefficients of the linear regressions were in the expected direction and close to significance. This may be due to the lower variance explained by PRS for intelligence compared to those explained by PRS for schizophrenia, a result already reported in the literature (liability *R*^2^ = 0.052 for IQ, *R*^2^ = 0.07 for schizophrenia) [10]. We used a detailed face-to-face assessment of the IQ, which may be more precise and homogeneous than the estimate in the largest cohort. PRS for intelligence was derived from a GWAS performed using the G factor, while PRS for educational attainment is derived from GWAS exploring the number of years the individuals have attended school, a proxy of intelligence. By using the LDSC method [42] we estimate the genetic correlation through the GWAS summary statistics between education attainment and cognitive performance to *r*_g_ = 0.68. Thus, the genetic variance of education attainment could be explained by cognitive ability but also non-cognitive skills, as proposed by recent study [43]. These methodological differences may have blurred the associations. Indeed, we found an association with PRS for cognitive performance in the general population which seems to be closer to the evaluations we performed. Due to the missing data in some subtests related to the change of WAIS versions, the working memory index could not be computed for all the individuals. Second, the follow-up duration is 1 year and we cannot exclude that some UHR-NC may have converted to psychosis afterwards. However, because the association between PRS for schizophrenia and FIQ is higher in UHR-NC, the misclassification of UHR-NC may have only limited the power of our study. Lastly, it has been reported that patients with schizophrenia have distinct trajectories of cognitive development. Indeed, three subtypes have been described: cognitively stable, preadolescent impairment and adolescent decline. Polygenic risk scores (PRS) were different between these three subtypes. We did not assess the cognition before the UHR phase and we might have mixed different subgroups of patients. Longitudinal assessment of cognition would be an interesting objective for another study.

Here, we explored the association between neurocognition in UHR individuals and PRS for various psychiatric disorders or phenotypic traits. We confirmed the impact of PRS for cognitive performance in UHR, the impact of PRS for schizophrenia, mostly in UHR-NC, and identified the protective role of PRS for resilience to schizophrenia on cognition of UHR. These results could help to identify individuals at higher risk of cognitive deficits but also patients that would benefit the most of cognitive training. Personalized approach based on PRS has been proposed in autism [44] and might be tested in early phases of psychosis. In addition, the genetic architecture of cognition in schizophrenia is not reducible to the genetics of schizophrenia. As cognitive deficit is important for social functioning, it deserves its own exploration to be able to identify new therapeutic strategies.

## Supplementary information

Supplementary Tables and Figures
