# Supplementary material for: Influence of polygenic risk scores for schizophrenia and resilience on the cognition of individuals at-risk for psychosis
Source: Transl Psychiatry. 2021 Oct 9;11:518. doi: 10.1038/s41398-021-01624-z (PMC8502171; doi:10.1038/s41398-021-01624-z)

**Supplementary Table 1**. Logistic regression of conversion status on PRS adjusted on age, sex and population structure

| **Conversion ~ PRS GWAS** | **GWAS SS*** | **P-val-T** | **ES*** | **SE*** | **R2** | **R2a** | **P** | **FDR P** |
| --- | --- | --- | --- | --- | --- | --- | --- | --- |
| Schizophrenia | 150 064 | 0.01 | 0.546 | 0.318 | 0.422 | 0.034 | 0.086 | 0.193 |
| Schizophrenia resilience | 66 617 | 0.0001 | 0.314 | 0.293 | 0.401 | 0.012 | 0.284 | 0.365 |
| ADHD | 55 374 | 0.05 | 0.171 | 0.278 | 0.393 | 0.004 | 0.537 | 0.537 |
| Bipolar disorder | 51 710 | 0.001 | 0.718 | 0.347 | 0.440 | 0.051 | **0.039** | 0.116 |
| Major depression | 480 359 | 0.0001 | 0.407 | 0.289 | 0.411 | 0.022 | 0.159 | 0.286 |
| Cross-disorder 2 | 727 126 | 0.2 | -0.725 | 0.342 | 0.440 | 0.051 | **0.034** | 0.116 |
| Intelligence | 269 867 | 0.1 | -0.885 | 0.408 | 0.447 | 0.059 | **0.030** | 0.116 |
| Educational attainment | 766 345 | 1.0E-08 | 0.243 | 0.286 | 0.396 | 0.008 | 0.395 | 0.444 |
| Cognitive performance | 257 828 | 0.4 | -0.339 | 0.314 | 0.402 | 0.013 | 0.279 | 0.365 |

GWAS SS: discovery sample size of the GWAS;

P-val-T: best fit p-value threshold;

ES: effect size (coefficient in the linear regression model after standardizing the variables);

SE: standard error;

R^2^a: adjusted R square, the variance explained by PRS;

ADHD: attention deficit hyperactivity disorder.

**Supplementary Table 2.** Linear regression of full WAIS IQ on PRS adjusted on age, sex and population ancestry

| **FIQ (<145) ~ PRS** | **GWAS SS*** | **P-val-T*** | **ES*** | **SE*** | **R^2^*** | **R^2^a*** | **P** |
| --- | --- | --- | --- | --- | --- | --- | --- |
| Schizophrenia | 150 064 | 0.1 | -0.225 | 0.118 | 0.153 | 0.035 | 0.060 |
| Schizophrenia resilience | 66 617 | 0.01 | 0.307 | 0.110 | 0.189 | 0.070 | 0.007 |
| Cognitive performance | 257 828 | 0.4 | 0.333 | 0.098 | 0.219 | 0.101 | 0.001 |

GWAS SS: discovery sample size of the GWAS;

P-val-T: best fit p-value threshold;

ES: effect size (coefficient in the linear regression model after standardizing the variables);

SE: standard error;

R^2^a: adjusted R square, the variance explained by PRS;

ADHD: attention deficit hyperactivity disorder.

**Supplementary Table 3**. Linear regression of PRS for schizophrenia, cognitive performance and resilience on the WAIS subtests and other cognitive tests

| **Cognitive traits** | **PRS_SCZ2** | | **PRS_cogP** | | **PRS_SCZ_resilience** | |
| --- | --- | --- | --- | --- | --- | --- |
|  | **ES*** | ***FDR***  ***P*** | **ES*** | ***FDR***  ***P*** | **ES*** | ***FDR***  ***P*** |
| ***WAIS score*** |  |  |  |  |  |  |
| PIQ | -0.289 | 0.089 | 0.242 | **0.040** | 0.268 | 0.068 |
| VIQ | -0.140 | 0.348 | 0.326 | **0.007** | 0.238 | 0.071 |
| ***Index scores*** |  |  |  |  |  |  |
| Verbal communication index | 0.120 | 0.305 | 0.399 | **<0.001** | 0.199 | 0.078 |
| Working memory index | -0.652 | **<0.001** | 0.525 | **0.019** | 0.456 | **0.007** |
| Perceptual organization index | -0.266 | 0.089 | 0.300 | **0.013** | 0.241 | 0.078 |
| ***Subtests scores*** |  |  |  |  |  |  |
| Arithmetic | -0.141 | 0.248 | 0.256 | **0.025** | 0.289 | **0.017** |
| Coding | -0.326 | **0.034** | -0.120 | 0.336 | 0.355 | **0.007** |
| Picture completion | -0.276 | 0.069 | -0.132 | 0.336 | -0.277 | **0.042** |
| Block design | -0.137 | 0.278 | 0.222 | 0.057 | 0.256 | 0.068 |
| Information | 0.200 | 0.097 | 0.302 | **0.007** | 0.254 | 0.061 |
| Matrix reasoning | -0.173 | 0.248 | 0.346 | **0.005** | 0.213 | 0.099 |
| Digit span | -0.476 | **<0.001** | -0.210 | 0.092 | 0.238 | 0.061 |
| Similarities | -0.118 | 0.380 | 0.418 | **<0.001** | 0.191 | 0.099 |
| Vocabulary | 0.151 | 0.248 | 0.325 | **0.009** | 0.134 | 0.240 |
| ***Other cognitive tests*** |  |  |  |  |  |  |
| D2 attention | -0.411 | **0.034** | 0.322 | 0.077 | 0.498 | **0.001** |
| verbal fluency semantic animals | 0.010 | 0.921 | 0.043 | 0.692 | 0.112 | 0.519 |
| verbal fluency phonologic letter p | 0.065 | 0.576 | 0.028 | 0.795 | 0.190 | 0.338 |
| TMT B minus TMT A | 0.343 | **0.007** | -0.238 | **0.040** | -0.168 | 0.111 |
| Stroop time denomination | 0.270 | 0.176 | -0.467 | **0.025** | -0.354 | **0.028** |
| WCST percentage perseverative errors | 0.163 | 0.252 | 0.107 | 0.336 | 0.371 | 0.060 |

ES: effect size (coefficient in the linear regression model after standardizing the variables).

**Supplementary Figure 1.** Linear regression of full WAIS IQ on PRS with 14 different GWAS P-value thresholds


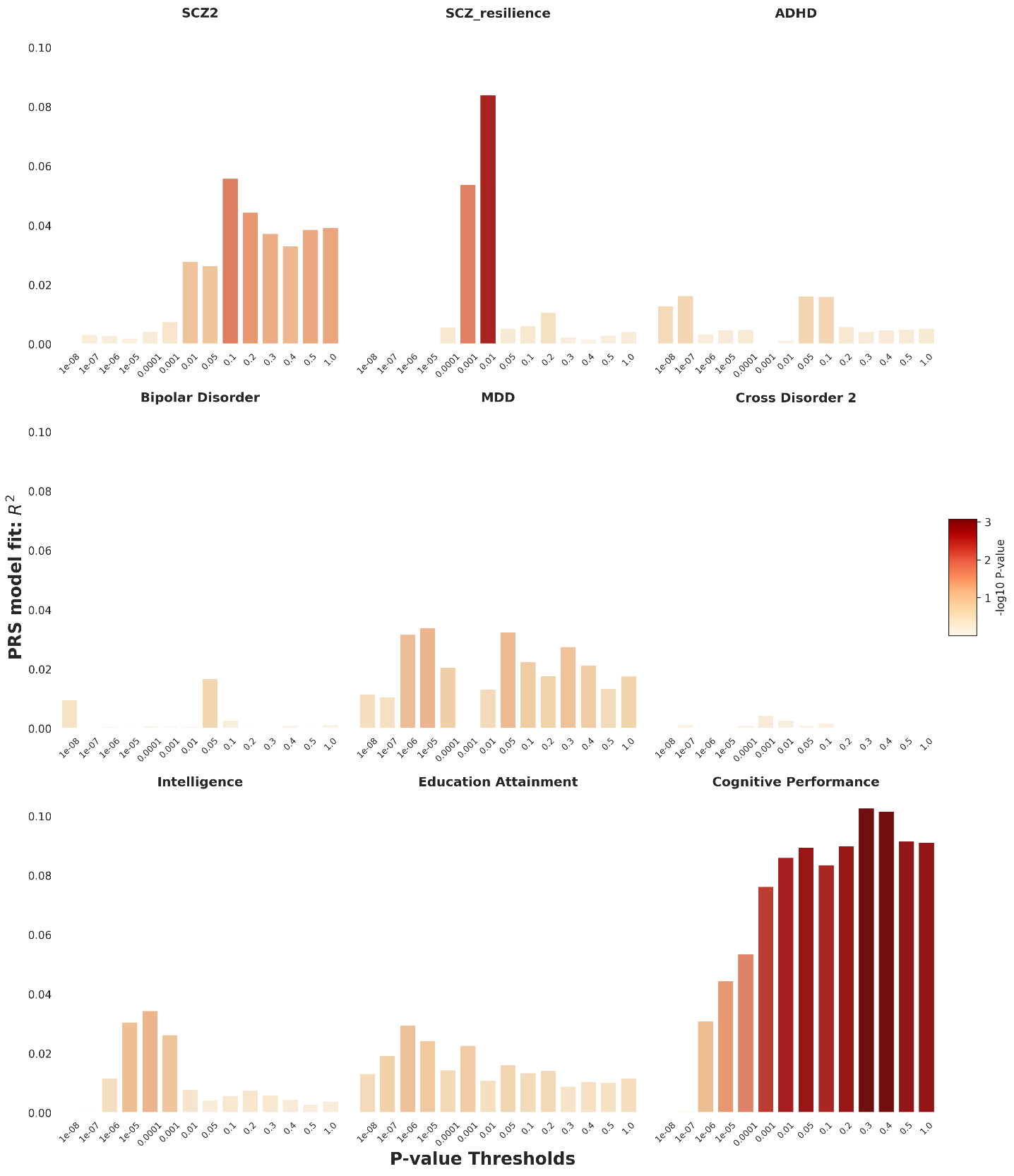


For the abbreviations: SCZ2: Schizophrenia; SCZ_resilience: Schizophrenia resilience; ADHD: attention deficit hyperactivity disorder; MDD: Major depression.

**Supplementary Figure 2.** Correlation plot of cognitive traits in the UHR cohort


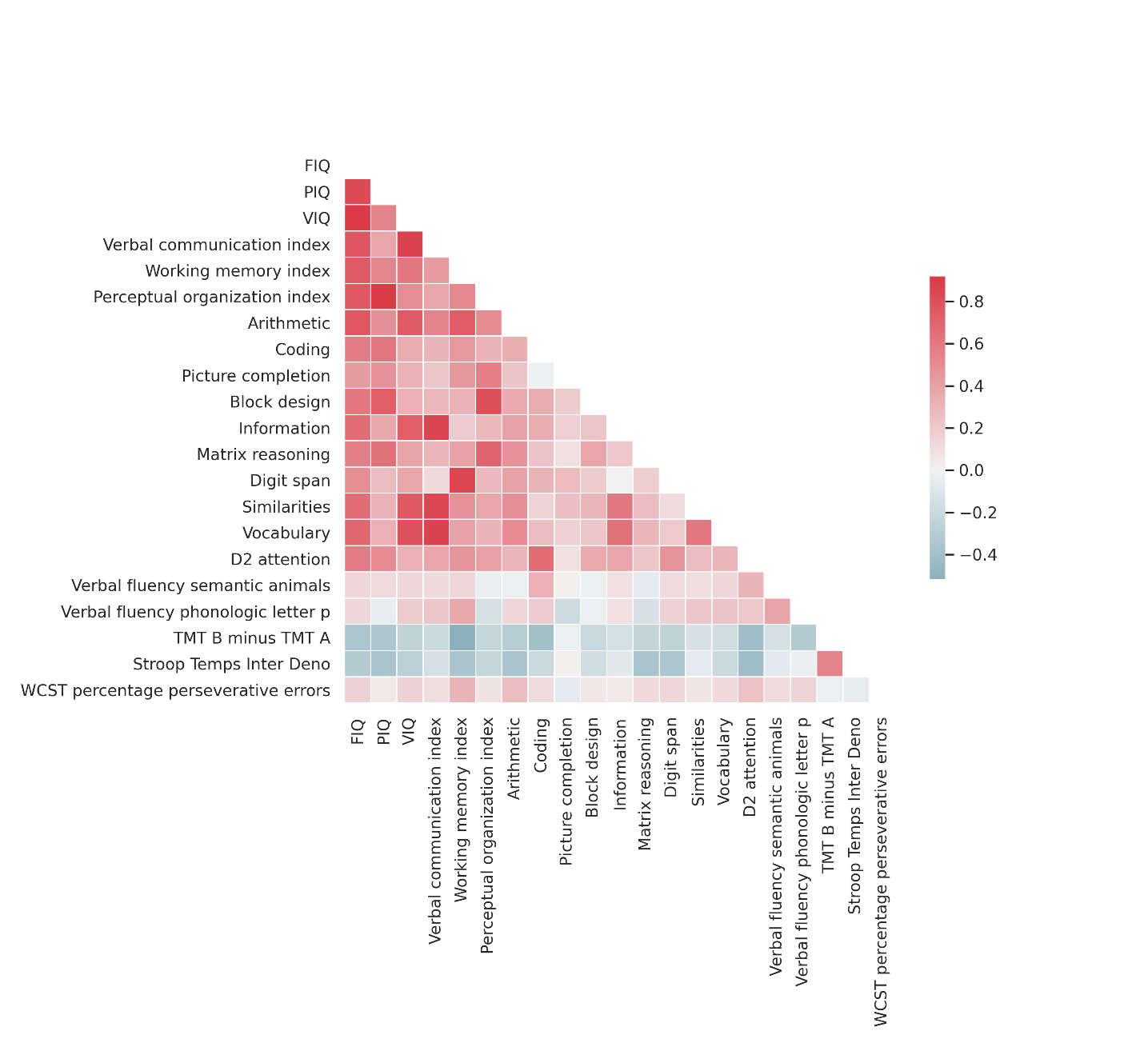

Supplement: Supplementary file 1 — Supplementary Tables and Figures [file 41398_2021_1624_MOESM1_ESM.docx]
